# Supplementary figures and images for: Mitochondrial ROS and HIF-1α signaling mediate synaptic plasticity in the critical period
Source: PLoS Biol. 2025 Aug 13;23(8):e3003338. doi: 10.1371/journal.pbio.3003338 (PMC12367176; doi:10.1371/journal.pbio.3003338)

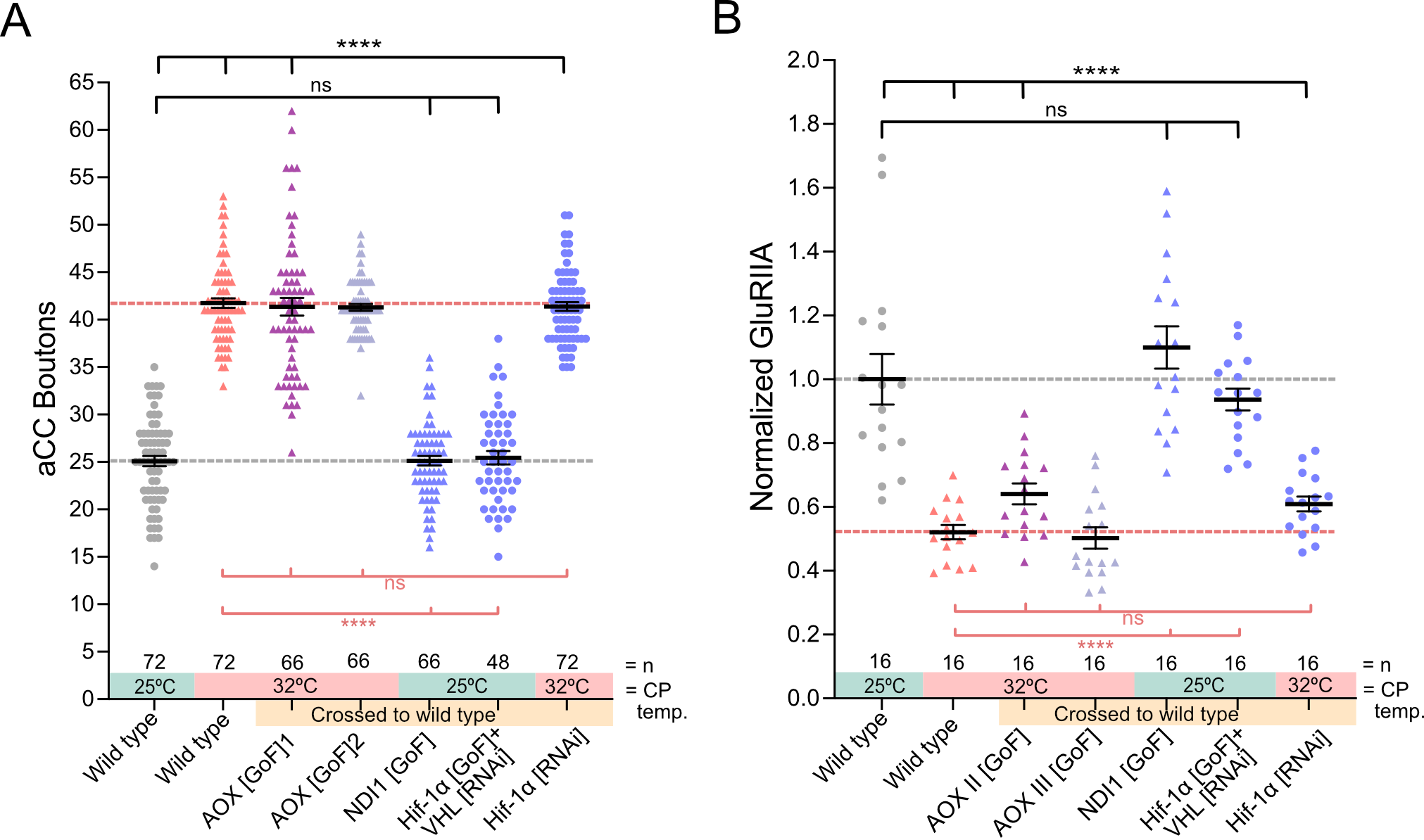

Supplement: S1 Fig — (A) Dot-plot quantification shows changes to aCC NMJ growth on its target muscle DA1, based on the standard measure of the number of boutons (swellings containing multiple presynaptic release sites/active zones). Data are shown with mean ± SEM, ANOVA, ****p < 0.00001, ‘ns’ indicates statistical non-significance. Black asterisks indicate comparison with the wild type of condition of 25°C throughout, genetically unmanipulated. Red asterisks indicate comparisons with wild type exposed to 32°C heat stress during the embryonic critical period. “Wild type” is Oregon-R. (B) Dot-plot quantification shows changes in levels of the GluRIIA receptor subunit at aCC NMJs quantified in C). Data are shown with mean ± SEM, ANOVA, ****p < 0.00001, ‘ns’ indicates statistical non-significance. Black asterisks indicate comparison with the wild type condition of 25°C throughout, genetically unmanipulated. Red asterisks indicate comparisons with wild type exposed to 32°C heat stress during the embryonic critical period. “Wild type” is Oregon-R. See raw data in S6 Data. (TIFF) [file pbio.3003338.s001.tiff]

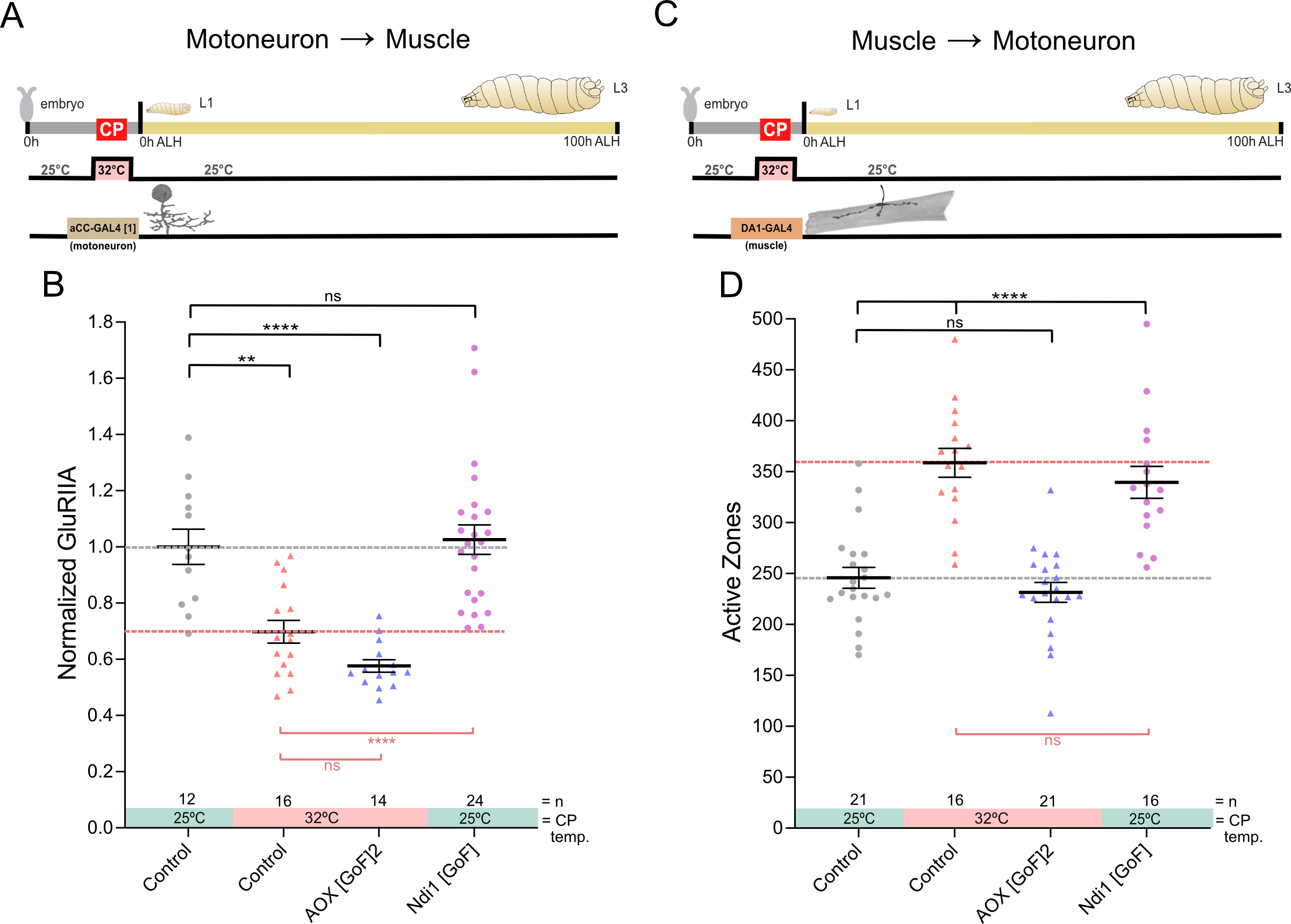

Supplement: S2 Fig — (A) Heat stress experienced during the embryonic critical period (32°C vs. 25°C control) leads to decreased postsynaptic GluRIIA. Simultaneous genetic manipulation of aCC motoneuron during embryonic stages does not affect GluRIIA in muscles. “Control” indicates control genotype heterozygous for Oregon-R and aCC-GAL4[1]. Larvae were reared at the control temperature of 25°C until the late wandering stage, 100 h after larval hatching (ALH). Data are shown with mean ± SEM, ANOVA, **p < 0.001, ****p < 0.00001, ‘ns’ indicates statistical non-significance. Black asterisks indicate comparison with the control condition of 25°C throughout, genetically unmanipulated. Red asterisks indicate comparisons with control genotype exposed to 32°C heat stress during the embryonic critical period. (B) Heat stress experienced during the embryonic critical period (32°C vs. 25°C control) leads to increased presynaptic active zone number. Simultaneous genetic manipulation of muscle DA1 during embryonic stages suggests that ROS by Complex-I is necessary and sufficient to induce active zones changes presynaptically. “Control” indicates control genotype heterozygous for Oregon-R and DA1-GAL4. Larvae were reared at the control temperature of 25°C until the late wandering stage, 100 h after larval hatching (ALH). Data are shown with mean ± SEM, ANOVA, ****p < 0.00001, ‘ns’ indicates statistical non-significance. Black asterisks indicate comparison with the control condition of 25°C throughout, genetically unmanipulated. Red asterisks indicate comparisons with control genotype exposed to 32°C heat stress during the embryonic critical period. See raw data in S7 Data. (TIFF) [file pbio.3003338.s002.tiff]

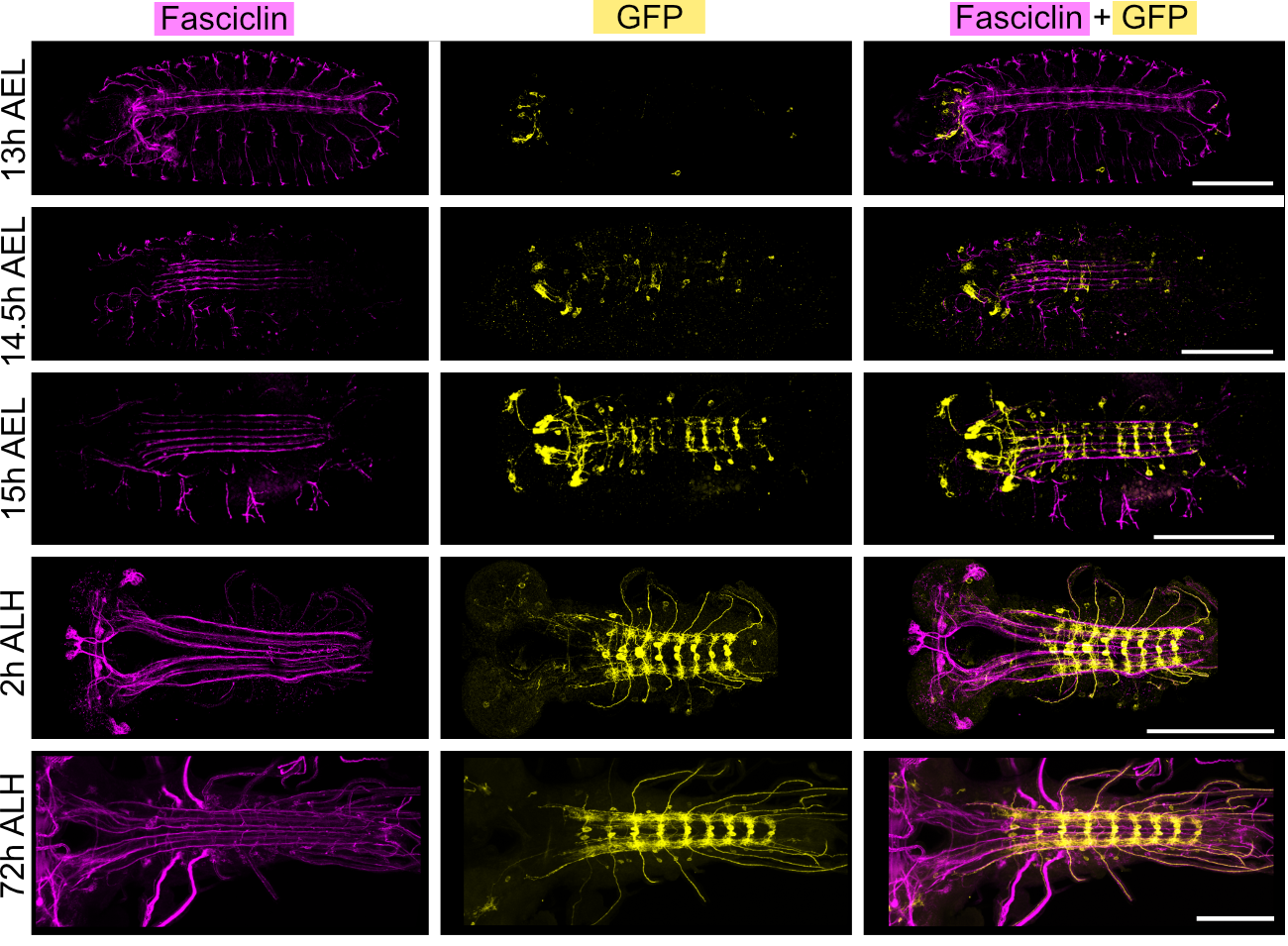

Supplement: S3 Fig — Expression starts around 13 h after egg lay (AEL) in few sporadic cells, not including the aCC motoneuron and mostly limited to the head region. First aCC motoneurons show GAL4 expression as of 14.5 h AEL, with more aCC motoneurons expressing from 15 h AEL onwards (i.e., 2 h prior to critical period opening). Concomitantly, GAL4 expression in other cells disappears. From laraval hatching onwards, segmentally repeated expression in all aCC motoneurons is maintained until at least 72 h after larval hatching (mid-3rd instar stage). Scale bar = 100 µm. (TIFF) [file pbio.3003338.s003.tiff]

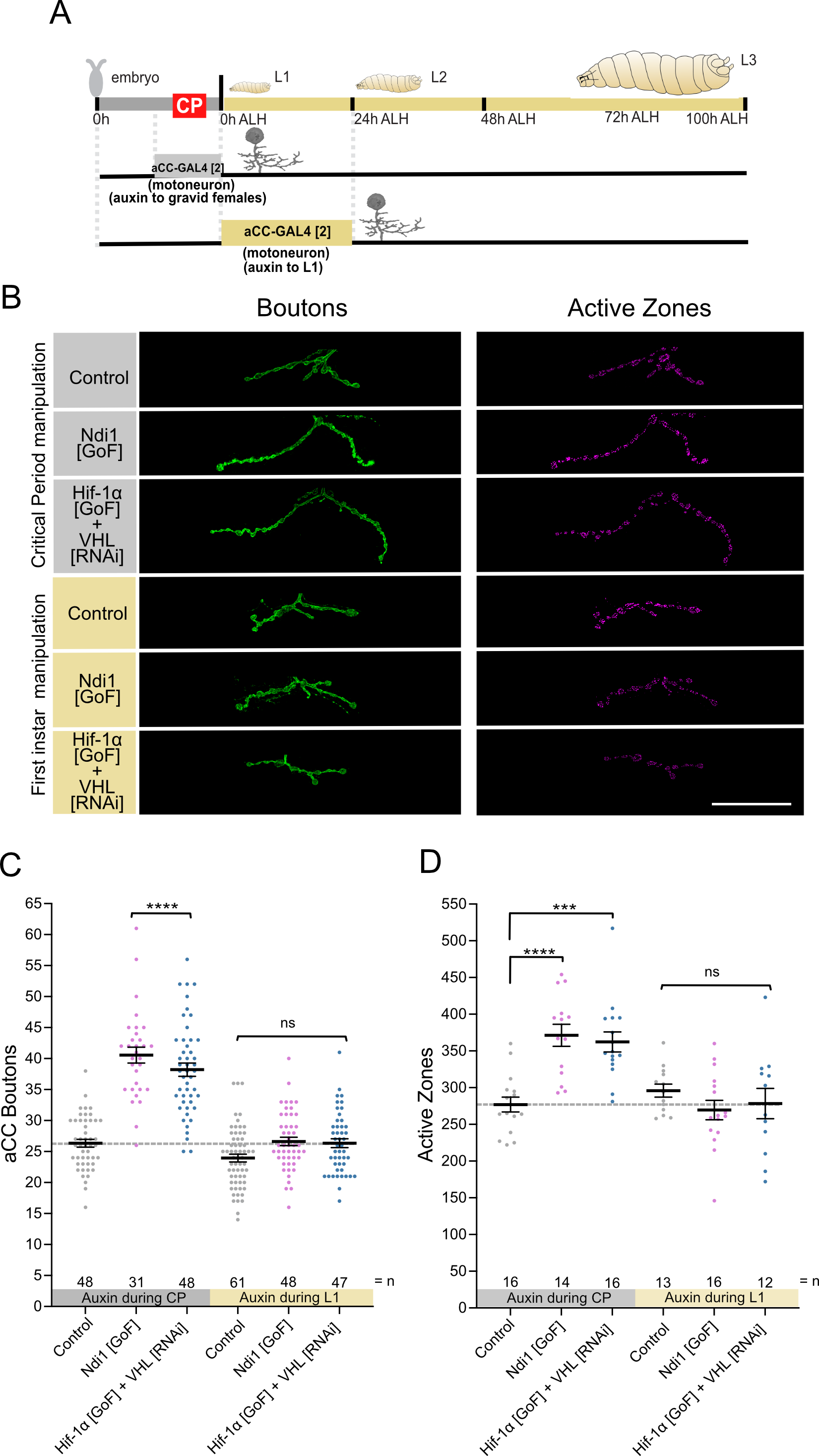

Supplement: S4 Fig — (A) Experimental paradigm. (B) Genetic manipulation of motoneuron aCC during the critical period versus after critical period closure. “Control” indicates control genotype heterozygous for Oregon-R and aCC-GAL4[2]; Auxin-GAL80. Larvae were reared at the control temperature of 25oC until the late wandering stage, 100 h ALH. Scale bar = 20 µm. (C) Dot-plot quantification shows changes to aCC NMJ growth on its target muscle DA1, based on the standard measure of the number of boutons (swellings containing multiple presynaptic release sites/active zones). Data are shown with mean ± SEM, ANOVA, ****p < 0.00001, ‘ns’ indicates statistical non-significance. (D) Dot-plot quantification shows changes in the number of active zones at aCC NMJs quantified in C). Data are shown with mean ± SEM, ANOVA, ***p < 0.0001, ****p < 0.00001, ‘ns’ indicates statistical non-significance. See raw data in S8 Data. (TIFF) [file pbio.3003338.s004.tiff]

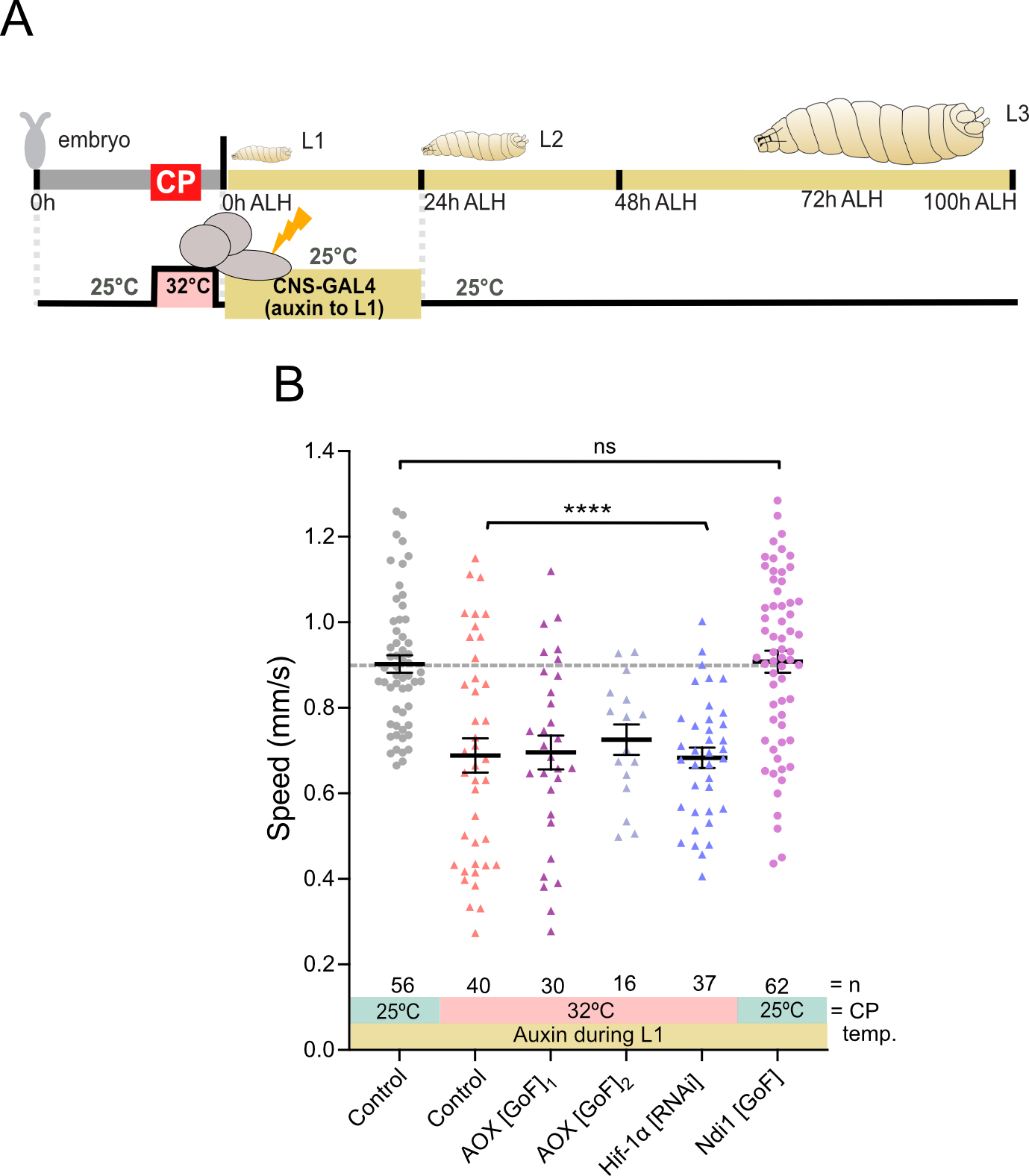

Supplement: S5 Fig — (A) Experimental paradigm. (B) Crawling speed of third instar larvae (72 h after larval hatching (ALH)). Temperature experienced during the embryonic critical period (25°C control vs. 32°C heat stress) and simultaneous genetic manipulation of all neurons during first instar stage. “Control” indicates control genotype heterozygous for Oregon-R and CNS-GAL4; Auxin-GAL80. Larvae were reared at the control temperature of 25°C until 72 h ALH. Each data point represents crawling speed from an individual uninterrupted continuous forward crawl, n = specimen replicate number, up to three crawls assayed for each larva. Mean ± SEM, ANOVA, ns = not significant, ****p < 0.00001. See raw in S9 Data. (TIFF) [file pbio.3003338.s005.tiff]

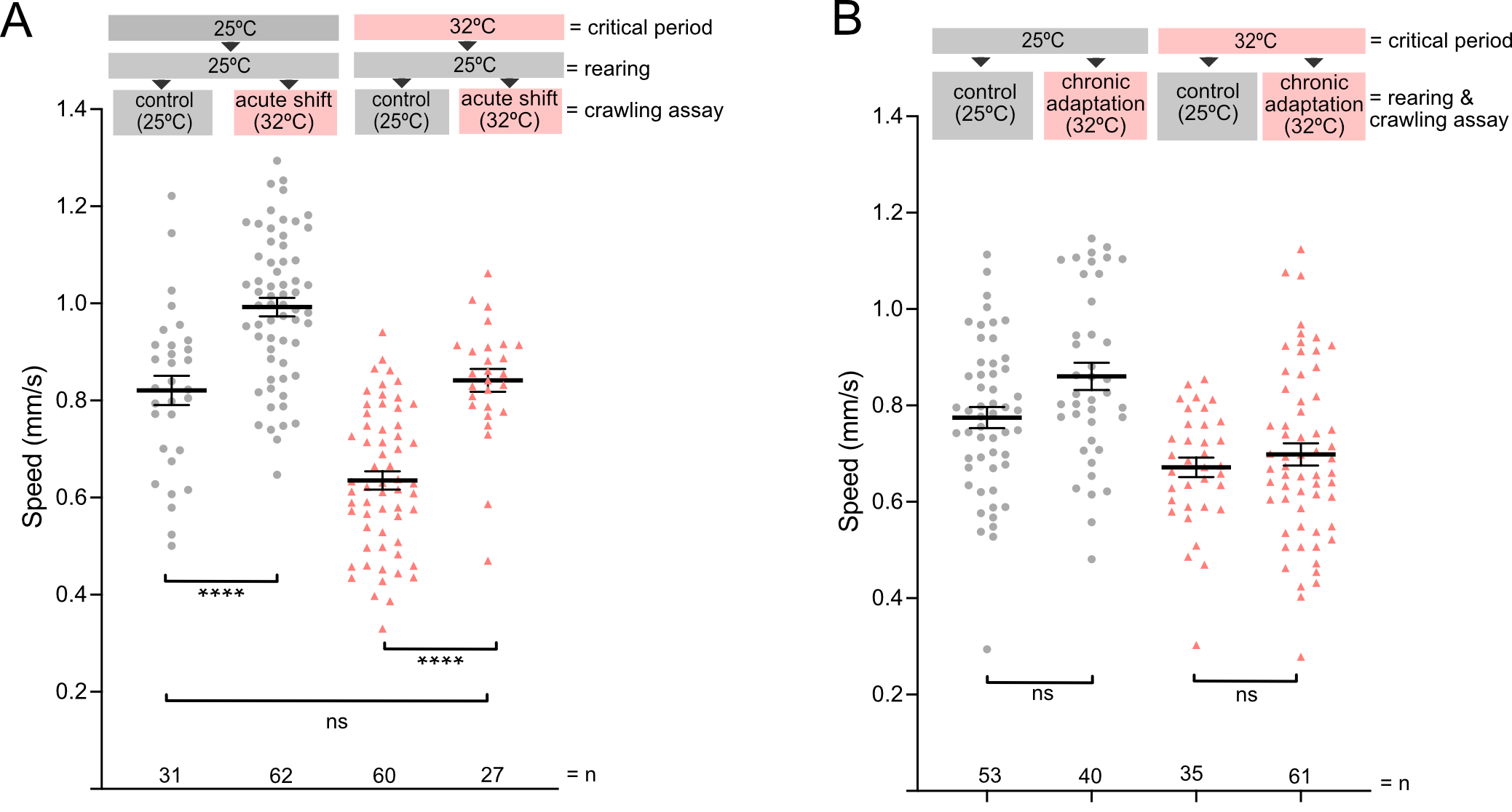

Supplement: S6 Fig — (A) Acute shift of temperature. Crawling speed of third instar larvae (72 h after larval hatching (ALH)). Temperature experienced during the embryonic critical period (25°C control vs. 32°C heat stress); temperature experienced during the larva rearing (25oC control vs. 32°C heat stress) and control (25°C) versus acute shift of temperature during the crawling assay (32oC). Genotype is Oregon-R. Each data point represents crawling speed from an individual uninterrupted continuous forward crawl, n = specimen replicate number, up to three crawls assayed for each larva. Mean ± SEM, ANOVA, ns = not significant, ****p < 0.00001. (B) Chronic adaptation to the heat stress. Crawling speed of third instar larvae (72 h after larval hatching (ALH)). Temperature experienced during the embryonic critical period (25°C control vs. 32°C heat stress); temperature experienced during the larva rearing and during the crawling assay (25°C control vs. 32°C chronic adaptation). Oregon-R. Each data point represents crawling speed from an individual uninterrupted continuous forward crawl, n = specimen replicate number, up to three crawls assayed for each larva. Mean ± SEM, ANOVA, ns = not significant. See raw data in S10 Data. (TIFF) [file pbio.3003338.s006.tiff]
