# Supplementary material for: Mitochondrial ROS and HIF-1α signaling mediate synaptic plasticity in the critical period
Source: PLoS Biol. 2025 Aug 13;23(8):e3003338. doi: 10.1371/journal.pbio.3003338 (PMC12367176; doi:10.1371/journal.pbio.3003338)
Supplement: Attachment — Submitted filename: Response to Reviewers.docx [file pbio.3003338.s019.docx]

We sincerely thank the reviewers for their insightful and constructive feedback on our manuscript. We value their positive evaluation of our work, including recognition of the novelty and significance of our findings regarding mitochondrial ROS and HIF-1α as mediators of critical period plasticity. We are especially grateful for the thoughtful suggestions, which have been invaluable. Through addressing those, these have strengthened the study.

Below, we provide a point-by-point response to each comment.

By way of an overview, here is a **summary of additional data generated and changes to Figures.**

**Figure 1**

- Added schematic of an NMJ (in A) indicating that this figure concerns postsynaptic muscle manipulations.
- Added a new panel (B) showing increased mitochondrial ROS levels in muscles following 32°C exposure at the end of embryogenesis.

**Figure 2**

- Added NMJ schematic (A) indicating this figure addresses postsynaptic muscle manipulations.
- Added a new group of experiments in C to E. Misexpression of NDI1 (Ndi1 [GoF]) in muscles DA1 together with exposure to 32ºC. Result shows not combinatorial (increased) effect when these 2 manipulations are done together vs independently.

**Figure 3**

- Added a new schematic of an aCC motoneuron (Figure 3A) to indicate that this figure addresses presynaptic motoneuron manipulations.
- Added new panel (B) showing increased mitochondrial reactive oxygen species (ROS) in motoneurons following 32ºC exposure during the critical period.
- Added a new group of experiments in D and E. Misexpression of NDI1 (Ndi1 [GoF]) in aCC motoneuron together with exposure to 32ºC. Result shows no combinatorial (increased) effect when these two manipulations are done together *vs* each independently.
- Added new panel (F) showing NMJ contact area. Results show increased NMJ area following transient exposure to 32ºC during the critical period and that this effect can be rescued by inhibiting ROS production in the mitochondria (AOX [GoF] or induced at 25ºC by increasing ROS in mitochondria (Ndi1 [GoF]).
- Added new panel (G) showing active zone density (active zones/NMJ area). Results show no changes in active zone density in any of the experimental conditions. This indicates, as expected, that following critical period manipulations active zone number scales with NMJ growth.

**Figure 4**

- Added a new NMJ schematic in (A) to indicate that this figure focuses on postsynaptic muscle manipulation.
- Added new panel (B) showing HIF1-alpha expression. Results show HIF1-alpha expression and localization in the nucleus of DA1 muscles following exposure to 32ºC or increase in ROS by mitochondria (Ndi1 [GoF]).

**Figure 5**

- Added new schematic representation in (A) of an aCC motoneuron indicating that panels (B-H) are about presynaptic motoneuron manipulations; and schematic representation of a VNC indicating that (K) addresses pan-neuronal manipulation of the VNC.
- Added new panel (B) showing HIF1-alpha expression. Results show HIF1-alpha expression and localisation to the nucleus of aCC motoneurons following exposure to 32ºC or increase in ROS by mitochondria (caused by Ndi1 [GoF]).
- Added new panels (C-E) showing results of HIF1-alpha manipulations in aCC motoneurons. Results show HIF1-alpha knockdown at 32ºC during the critical period rescues the effect of temperature; while increasing HIF1-alpha at 25ºC during embryogenesis mimics the effect of 32ºC heat stress.
- In (F) we added one example of a trace.
- Added new panel (I): Quantification of the first action potential amplitude (in mV) recorded from aCC motoneurons under different experimental conditions. Results show no significant changes between groups.
- Added new panel (J): Duration of first action potential fired (measured at 50% relative amplitude). Results show no significant differences between groups.

**Supplementary figure 1 (new figure)**

- Additional controls for experiments in Figure 1. UAS lines crossed to wild type show that observed effects are dependent on GAL4-driven expression during the critical period and therefore specific to RNAi targets.

**Supplementary figure 2 (new figure)**

- 2A: aCC motoneuron manipulations during the embryogenesis do not have a lasting impact on GluRII A in muscles.
- 2B: DA1 muscle manipulations during the embryogenesis have a lasting impact on active zones number.

**Supplementary figure 3 (new figure)**

- Characterization of the time and location aCC-GAL4 [2] line. Results show sparse, irregular pattern of a few cells expressing GAL4 at 13 hours after egg laying; these differ between segments and none represent the aCC motoneuron. At 14.5 hours after egg laying (late stage 16) a few aCC motoneurons express GAL4, while from 15 hours after egg laying onwards there is clear, segmentally repeated expression, almost exclusively, in aCC motoneurons. This is maintained thereafter, as probed at larval hatching and the mid-third instar stage.

**Supplementary figure 4 (previously supplementary figure 1)**

- Added a schematic of an aCC motoneuron (A) to indicate that the figure concerns presynaptic motoneuron manipulations.

**Supplementary figure 5 (previously supplementary figure 2)**

- Added new schematic representation of a VNC indicating that this figure is about manipulations in all neurons.

**Supplementary figure 6 (previously supplementary figure 3)**

*No changes*

Reviewer #1 (James Jepson)

*Overview: 
In this work, Sobrido-Cameán and colleagues investigate the molecular mechanisms underlying a critical period of sensitivity to heat stress during the development of the Drosophila larval locomotor network. 
The authors show that heat stress during a narrow window of developmental time (17-19 h after egg laying) causes long-lasting alterations in pre- and post-synaptic development and neural excitability during the larval stages of the Drosophila lifecycle. Using elegant genetic approaches, they identify mitochondrial reactive oxygen species (ROS) as a key mediator of these alterations. Furthermore, they provide evidence that the conserved epigenetic regulator HIF-1alpha/Sima acts downstream of ROS to drive these long-term changes in development and intrinsic excitability.*

*The identification of ROS and HIF-1alpha as effectors of a critical period influencing locomotion is both novel and exciting, and may help further elucidate the molecular underpinnings of critical periods during neural development. Since these developmental phases are disrupted in a range of neurological disorders, this work both advances the fundamental understanding of neural development and has potential clinical relevance.*

*The studies themselves are well performed and include technically challenging electrophysiological recordings from larval motoneurons. There are certain controls that could be added to improve robustness. Additional analyses of existing data and textual alterations could provide further mechanistic insights and help the readers understand the work more clearly. Finally, it would be highly beneficial if the authors could directly test for increases in pre- and post-synaptic ROS in response to heat stress, as predicted by their model and prior data. With these inclusions, I would be very happy to see this interesting work published in PLoS Biology.*

*Major comments:*

*1. ROS levels.*

*The authors use several independent genetic manipulations to show that ROS is required post-synaptically for heat-induced changes in post-synaptic GluRIIA levels, and presynaptic bouton number (Figure 1), pre-synaptically for heat-induced changes in presynaptic bouton number (Figure 3), and pre-synaptically for heat-induced changes in intrinsic motoneuron excitability (Figure 5). The authors mention prior work showing that ROS can mediate activity-dependent synaptic growth (a presynaptic effect), and that heat stress elevates ROS (page 3). However, they do not directly show that in their experimental paradigm, embryonic heat stress increases ROS, and importantly, whether this occur both pre- and post-synaptically (as predicted).*

*I agree with the authors that this is likely to occur, but if it did not, it would significantly alter their mechanistic model. Therefore, it would be beneficial for the authors to test whether ROS is indeed elevated by heat stress during the embryonic critical period, in motoneurons and the post-synaptic muscle tissue, perhaps using transgenic reporters such as UAS-mito-roGFP2-Orp1, as they have used previously (Oswald et al., (2018) eLife).*

In our opinion, the genetic phenotypic data are clear and entirely consistent: the many different manipulations carried out implicate mitochondrial ROS as required and sufficient for the NMJ phenotypes caused by embryonic critical period heat stress. Perhaps most directly among the five different manipulations are rescue by mitochondrial-targeted catalase as well as alternative oxidase (AOX). Conversely, increasing mitochondrial ROS levels independently of temperature is sufficient to recapitulate the phenotype, highlighting the instructive role of ROS during the critical period. Nonetheless, we agree that a direct measure of changes in ROS levels under the specific experimental conditions, e.g. in response to heat stress, would be an important addition. We have therefore conducted additional experiments using a genetically encoded redox sensor, similar to the roGFP2-Orp1 probe suggested by the reviewer.

In our experience, the roGFP2-Orp1 ROS sensor had serious limitations as a genetically encoded ROS sensor; in terms of expression level, sensitivity and limited dynamic range (see Oswald et al. (2018), Figure 2A). The Tobias Dick lab, who had initially published the roGFP2-Orp1 ROS sensor in 2011 had since developed a greatly improved version (Morgan et al., 2016), for which we generated new transgenic Drosophila stocks: 10xUAS-IVS-mito::roGFP2::Tsa2ΔCPΔCR. As detailed in the original publication, Tsa2 is a typical 2-Cys peroxiredoxin with rapid and selective reactivity toward H₂O₂. The ΔCR mutation eliminates the resolving cysteine, preventing thioredoxin-mediated reduction and thereby enhancing sensitivity by stabilizing the oxidized state. Compared to roGFP2-Orp1 or previous HyPer versions, this probe was reported to offer:

- at least 20-fold higher sensitivity to H₂O₂
- minimal pH sensitivity in physiological ranges (issues with earlier HyPer variants)
- high specificity for peroxides like H₂O₂ and tert-butyl hydroperoxide
- stoichiometric response to H₂O₂, with linear and quantitative oxidation dynamics

We targeted this probe to mitochondria and expressed it in both motoneurons and muscle tissue using tissue-specific GAL4 drivers. Using this tool, we found a significant and robust increase in mitochondrial ROS levels in embryos exposed to 32ºC during the critical period, in both neurons and body wall muscles. These results directly demonstrate that embryonic heat stress elevates mitochondrial ROS in these cells. We have now included these new results in the revised manuscript (see new Figure 1B and Figure 3B) and detailed them in the Results section.

*2. Methods for transient transgene expression.*

*The schematics in Figures 1-6 depict transient control of transgene expression during (approximately) the embryonic critical period. How this is achieved is not immediately clear to the reader - the AGES system is only described after Fig. 3B-D are mentioned (for Supp. Fig. 1), and in the legend of Fig. 5. Can the authors please clearly explain to the reader how they control transgene activity at the earliest stage (i.e for Figure 1), and mention the logic of using the AGES system? (I presume this was used because the more commonly used TARGET system requires temperature changes?).*

*This leads to an important control condition that appears to be currently missing in some datasets - an auxin-fed transgene or driver-alone control containing auxin-Gal80. This genotype appears present in Figure 5 and Supp. Figs 1 and 2, but it was not clear to me whether this control was also fed auxin at the same time as the experimental genotypes (apologies if I missed it). Please clarify in the text and add this control to relevant datasets if absent.*

The GAL4 expression lines that we use to target gene expression selectively to a single muscle per hemisegment (SS2-C-GAL4 [referred to as DA1-GAL4]) or a specific motoneuron (RN2-O-GAL4 [referred to as aCC-GAL4-1]) happen to express GAL4 only during the embryonic stage. We confirmed that once the animal hatches, GAL4 expression ceases. These transgenic lines were generated by Miki Fujioka. Both utilise enhancer fragments from the *even-skipped* gene, and a separate *eve* regulatory element is required to maintain expression into larval stages. Therefore, when using these lines, we did not need auxin-GAL80. We have included this as “additional information in the Table 1.

To test the importance of critical period timing, we needed to perform equivalent manipulations at a different stage of development, after the critical period had closed. This we achieved using an independently generated transgenic line that expresses GAL4 in the aCC motoneuron continuously (GMR94G06-GAL4), but where the activity of GAL4 can be temporarily controlled. We achieve this through the AGES system, which controls GAL4 activity via an auxin-sensitive GAL80 (GAL4 repressor). Importantly for this study, the AGES system is controlled independently of temperature (traditionally one would use temperature-sensitive GAL80).

In doing so, we firstly demonstrated that any phenotypes generated via RN2-O-GAL4/aCC-GAL4[1]-based manipulations were reproduced when using an independently generated aCC-GAL4 line, i.e. independent of the particular GAL4 line, and specific to the manipulation during the embryonic critical period (the same manipulations carried out after critical period had no lasting impact).

As an interesting detail, a further advantage of using the GMR94G06-GAL4 (aCC-GAL4) line is that it activates GAL4 expression later than RN2-O-GAL4, starting around 14.5 hours after egg laying, i.e. 2.5 hours before critical period opening, at a time when aCC has fully differentiated and initiates synapse formation: the aCC growth cone has begun to form a neuromuscular junction at its target muscle, DA1, and dendrites have formed centrally, with synapses being formed on those. We have now added a supplementary figure (Supplementary Figure 3) to show the window of GAL4 activity.

For behavioural tests, the entire nervous system needed to be manipulated in a temporally controlled manner. So, we combined an n-Syb-GAL4 transgene (GMR57C10-GAL4) with the AGES system/auxin-sensitive GAL4 repressor.

The control condition “auxin-fed transgene with driver-alone”, which contains auxin-GAL80, is the “Control” group in the mentioned figures. We apologize for not stating this more clearly. We now changed the figure legends to explain this more clearly.

*3. RNAi line controls.*

*The authors use various RNAi lines (#26207, 50727, 34717) that are in a y, sc, v, sev quadruple mutant background (Figures 4-5). Have the authors tested RNAi alone controls to confirm that synaptic development is not altered in male hemizygotes or heterozygote females for these mutants?* 
We always use males from the UAS-RNAi lines. The above mutations are mostly on the X chromosome, i.e. no offspring would ever be homozygous for those, but at best heterozygous. If there was an issue, we would expect a bimodal data distribution, which we have not seen; nor have there been indications of phenotypic changes, when we brought such RNAi lines into a different genetic background via recombination or standard genetic crosses (resulting in a w1118 X-chromosome background). In summary, we have not seen indications that the genetic background has a significant impact on the critical period phenotypes we documented.

Regardless, we agree with the reviewer about the importance of that controls. We have tested the RNAi lines crossed to wild type flies (Supplementary Figures 1 and 2). None present with any phenotypic effects, demonstrating that effects observed only occur in the context of GAL4 expression, and only when this takes place during the critical period.

*4. Morphological analyses at the NMJ.*

*At the larval NMJ, the authors focus on the effect of heat stress on presynaptic bouton number and post-synaptic GluR expression. The images provided by the authors generated questions to me about two other aspects of NMJ development. Firstly, whether heat stress also affects bouton size? This seems to decrease somewhat in the 32C control, but unlike bouton number, this property does not seem altered by AOX over-expression (at least, in the image provided; Fig. 1B). This raises the question of whether some morphological alterations are driven by ROS, but others are ROS-independent? Secondly, have the authors quantified ghost boutons at the NMJ (PMID: 18341991), and tested whether their number is affected by heat stress? Again, images in Fig. 1B are suggestive of potential alterations. A decrease in ghost bouton number might suggest a pathway to increase mature bouton number.*

*These suggestions are not essential to address but could yield interesting mechanistic insights.*

We did not observe a significant number of ghost boutons, if any, in any of the conditions tested. This might be partly linked to the fact that the NMJ on muscle DA1 is much smaller than the one on muscles 6/7 that are commonly used for assessing ghost boutons; and that the DA1 NMJ generates ghost boutons at a correspondingly much lower frequency (*own* *unpublished observations*).

Regarding bouton sizes, we explored this, and analysed with lumping into different size bins. The data are noisy and not very informative. We have therefore focused on bouton number (as the most reproducible indicator of NMJ size) and overall NMJ-muscle contact area (the next best measure).

We found that changes in NMJ contact area reflect changes in bouton number, i.e. both are measures of NMJ size (data now included in Figure 3F).

We used bouton number as our primary readout, because it is the more robust and reliable indicator of phenotypic differences, and therefore also the most commonly used in the field.

*5. Presynaptic and postsynaptic effects of ROS/HIF-1alpha.*

*The authors identify a mix of pre- and post-synaptic requirements for ROS and HIF-1alpha in driving heat-induced changes in development during the critical period. ROS are required post-synaptically for changes in bouton number and GluRIIA levels Fig.1), and pre-synaptically for increased bouton and active zone number (Fig.3) and reduced intrinsic excitability (Fig.5); while HIF1-alpha is required post-synaptically for changes in bouton number and GluRIIA levels (Fig.4), and pre-synaptically for reduced intrinsic excitability (Fig.5).*

*These complex requirements emphasize the importance of determining where ROS is increasing (see above) in response to heat stress. The results also suggest that post-synaptic ROS/HIF1-alpha induce retrograde signals that impact presynaptic bouton number. However, it is unclear whether the reverse is true: can enhancing presynaptic ROS/HIF1-alpha act trans-synaptically to alter post-synaptic GluRIIA levels?*

We agree that the combined pre- and postsynaptic effects suggest complex communication across the synapse, which is expected and has been studied extensively, e.g. around TGF-ß and Wnt signalling. It is possible that the manipulations we have carried out impact on these or other pathways; though that would be a different study in its own right.

We observed that the development of neuromuscular junction size is subject to both muscle- as well as motoneuron-targeted manipulations during late embryogenesis. Manipulation of either partner cell during the embryonic critical period can cause a subsequent increase in NMJ size by the late third instar stage; or, conversely, rescue such an overgrowth in a systemically manipulated animal (e.g. by heat stress). The number of presynaptic release sites (active zones) scales with NMJ size, as a function of this overgrowth. This is unlike homeostatic reductions, which occur after prolonged overactivation of the motoneuron (as per Oswald et al., 2018).

Importantly, there are distinction between muscle vs neuron critical period manipulations. Muscle-targeted manipulations also alter the composition of the postsynaptic GluRII receptor patches. In contrast, motoneuron-targeted critical period manipulations do not affect the GluRII composition, suggesting that this feature is exclusively directed by the postsynaptic muscle (Supplementary Figure 2). This asymmetry may reflect a temporal sequence or hierarchy in critical period plasticity: during embryogenesis, body wall muscles become electrically active before motoneurons can conduct action potentials. This timing could allow muscles to undergo their critical period and establish receptor composition before motoneurons can influence this aspect of synaptic development. We have added this consideration to the discussion.

*In the context of temperature-induced changes in larval crawling, the above results also made we wonder whether the authors had tested how muscle-specific manipulations of AOX/HIF1 affected crawling speed; and/or whether the authors had distinguished whether this pathway was relevant solely in motoneurons (using a motoneuron-specific driver) for adaptations to heat stress, or were required more broadly throughout the CNS?*

*Again, these are not essential experiments but their inclusion might help more narrowly define relevant domains in which the ROS/HIF1 axis is important during the critical period.* 

We apologize for not explaining this more clearly. To assess the behavioural effects, we performed pan-neuronal manipulations using the R57C10-GAL4 (in effect an n-Syb-GAL4 line).

None of the single-cell manipulations affected behaviour. For muscle-specific manipulations, we targeted the DA1 muscle and, as the reviewer correctly pointed out, there was no effect on crawling behaviour since only one muscle was manipulated. A key strength of our approach are single-cell manipulations. Using the SS2-GAL4, we specifically manipulate the DA1 muscle, and with the RN2-O-GAL4, we target only the aCC (and RP2) motoneuron(s). This allows us to avoid impacting the animal more broadly, e.g. its feeding behaviour, crawling activity, etc. This cell-specific approach is essential to clearly identify cell autonomous mechanisms and cause-effect relationships.

We showed that the same mechanism (the RET-ROS – HIF1 axis) that regulates NMJ development also affects behaviour, by manipulating the same pathway within the entire nervous system. Separately, we have some data that indicate that manipulations of all muscles do not affect crawling speed (not shown). To selectively manipulate all motoneurons is not straight forward, as no GAL4 line that I am aware is sufficiently specific to exclude spilling over into more network-wide effects.

We have revised the text to improve clarity and to ensure that our experimental approach is fully explained.

*6. Basis of reduced intrinsic excitability (Figure 5).*

*The authors show that heat stress during the critical period reduced AP firing rate without altering RMP. How do the authors stipulate that AP frequency is being altered? Could the authors examine AP shapes in their datasets to test whether this property is affected by embryonic heat stress? For example, could the duration/amplitude of the afterhyperpolarization current be increased? Or spike width?*

We carried out further analysis of action potentials. Focusing on the first action potential for each recording between groups, we saw no statistically significant differences between groups in either action potential amplitude (Figure 5I) or duration, quantified as the full width at half maximum (FWHM) amplitude (Figure 5J). Though the caveat is that these are not the most sensitive measures, especially in Drosophila where the action potentials are generated far away from the site of recording.

*Minor comments/questions:*

*1.  How do the authors know that feeding auxin to gravid females induces transgene expression during the latter half of the embryonic stage? (Supp. Fig. 1A).*

To determine that feeding auxin to gravid females induces transgene expression during the latter half of the embryonic stage, we tested the GAL4 expression using a fluorescent reporter to precisely time the GAL4 activation.

The phenotypes demonstrate that feeding auxin to gravid females induces transgene expression during the latter half of embryogenesis, as these mimic the effects of direct GAL4 expression (e.g. of RN2-O-GAL4). Importantly, these phenotypes only occur when auxin is fed to gravid females. They do not occur post critical period induction of GAL4 activity, e.g. when auxin is fed to freshly hatched larvae. We have now clarified this in the text.

*2. Descriptions of manipulations. E.g Fig.5, p.6. For all figures, can the authors please specific in the text whether transgene manipulation is occurring pre- or post-synaptically? i.e the effect is cell-autonomous or non-cell-autonomous.*

We agree with your suggestion and apologize for not making this clearer. We have revised the text throughout the manuscript to explicitly specify whether transgene manipulations are occurring pre- or post-synaptically.

*3. Figure 5: it was unclear to me why HIF-1alpha and VHL manipulations are combined here (Fig.5C-D) but not elsewhere in the text. Can the authors please clarify and provide rationale?*

The reason we combined HIF-1α and VHL manipulations in Figures 5C-J was to achieve a more robust manipulation of HIF-1α activation during the critical period. HIF-1α is constantly expressed in cells but also continuously degraded under normal conditions; so its levels are tightly regulated, even when mis-expressing HIF-1α [GoF]. In our previous experiments, we showed that overexpressing HIF-1α [GoF] (as shown in Figure 4) leads to specific phenotypic effects, and similarly, knocking down VHL (as shown in Figure 4) also causes a significant impact due to impaired degradation of HIF-1α. When designing the electrophysiological experiments, we aimed to focus on key manipulations to minimize the number of conditions in light of the challenge of carrying out those difficult measurements, and therefore seeking to avoid excessive experimental work. Therefore, to induce a sustained, significant effect on HIF-1α, we chose to combine the HIF-1α [GoF] overexpression with VHL [RNAi], which reduces the effectiveness of HIF-1α degradation. This combined manipulation allowed us to achieve a more consistent induction of HIF-1α levels during the critical period while keeping the experiment manageable. We added an explanation on this to the text.

*4. Please define the 'CNS-Gal4' - I presume this is R57C10-Gal4 (Table 1), but I could not find a statement to this effect.*

We apologize for the oversight. We have now updated the text to clearly define 'CNS-GAL4' as R57C10-GAL4. Thank you for bringing this to our attention.

*5. Discussion: the authors state they 'demonstrate the cell-autonomous necessity of RET-generated mitochondrial ROS and HIF-1a signalling for heat stress-induced critical period plasticity' (p.8). However, many effects described in this manuscript are non-cell-autonomous (e.g retrograde changes at the NMJ). Can the authors clarify that some of the effects are cell-autonomous, whereas others are non-cell-autonomous?*

We agree with the reviewer. Some of the effects described in the manuscript are indeed cell-autonomous, while others, such as the retrograde changes at the NMJ, are non-cell-autonomous. We have modified the discussion accordingly to better clarify.

Reviewer #2

*Sobrido-Cameán et al. demonstrate that temperature stress during the late embryonic critical period (CP) induces synaptic plasticity at the Drosophila neuromuscular junction. Using mutant lines and analyses of presynaptic morphology, glutamate receptor expression, synaptic active zones, electrophysiology, and crawling behavior, they reveal that CP plasticity is driven by ROS generation via reverse electron transfer, leading to HIF1-alpha induction.*

*Comments:*

*-	The legend of figure 4 is missing and seems to be copy-pasted from figure 2.*

We apologize for the oversight regarding the figure legend in Figure 4. We have updated the legend to ensure it accurately corresponds to the figure.

*-	A recent study (PMID: 39721493) showed onset of presynaptic plasticity after acute emission of mitochondrial hydrogen peroxide in late-stage larvae. The authors should cite this work and discuss how it relates to their finding that RET-ROS induces plasticity if induced during but not after the critical period (supp fig 1).*

Thank you very much for pointing out this reference, which had escaped our attention. We have added it to the text.

Our previous studies investigated the effects of chronic manipulations of neuronal activity and/or mitochondrial ROS throughout larval development, after closing of the embryonic critical period. The more recent study by Irina Stavrovskaya and colleagues, is a much-needed advance on this, using short-term acute stimulation and/or ROS generation. However, in that study, the expression of cytoplasmic catalase was chronic and spanned developmental stages, including the critical period. In contrast, our current findings demonstrate that transient ROS production at mitochondrial complex I, specifically during the critical period, but not after, induces lasting structural plasticity (see Supplementary Figure 4), highlighting the importance of developmental timing in redox-mediated synaptic modulation.

Reviewer #3

*The importance of critical periods during neurodevelopment cannot be understated; disruption of synaptic activity during developmental critical periods has lasting effects on synaptic morphology, growth, function, and behavioral coordination. Though the existence of critical periods has long been known, the events that occur during those critical periods and importantly, the molecules needed to enact them have remained considerably more mysterious. Sobrido-Cameán and colleagues here identify two fundamental new concepts regarding critical period biology: first, that manipulating a single cell during a critical period can influence later changes in morphology, protein recruitment, and activity; and second, that reactive oxygen species (ROS) from mitochondria and acting through HIF-1α are required to mediate critical period signals. Mitochondrial ROS is required to signal in postsynaptic muscles or presynaptic motoneurons - in the absence of ROS (either through failure to generate or subsequent degradation), critical period changes induced by high temperature bouts are blocked. These changes are seen morphologically (involving bouton number, GluRIIA abundance, or active zone number) and functionally (involving action potential number or crawling ability. In muscles, blocking (either by directly impairing or expressing enzymes that regulate its levels) HIF-1α precludes ability to induce changes during the critical period.*

*The findings represent a significant advance in our understanding of the molecular basis of critical period signaling. Moreover, the data posit a fascinating new addition to the burgeoning concept that ROS are not just a deleterious byproduct of existence, but rather, an important neuronal signaling axis. The experiments are particularly well designed and the genetics very well controlled, with examples of loss-of-function and gain-of-function consistent with each other. Moreover, the use of exogenously applied constructs (like yeast Ndi1 and AOX) to demonstrate similar manipulations of ROS is particularly helpful. There are, however, a few points that should be addressed in a revised manuscript.*

*Major Issues:*

*1)	Does this work transsynaptically? When mitochondrial ROS is manipulated in motoneurons, does this influence GluRIIA abundance? When mitochondrial ROS or HIF-1α is manipulated in muscle, does this influence active zones? Similarly (if possible), does concurrent transsynaptic manipulation enhance the effects during the critical period or does one cell basically hit the ceiling of how much the system can change?*

Briefly, we found that manipulating mitochondrial ROS in motoneurons does not affect postsynaptic GluRIIA abundance, which is regulated by muscles during their earlier critical period. However, when mitochondrial ROS are manipulated in muscles, it does cause subsequent NMJ overgrowth with presynaptic active zone number scaling proportionately. We interpret this as both motoneuron and muscle being able to direct overall growth of the presynaptic terminal, but the earlier phasing of the muscle critical period prevents motoneurons from influencing the muscle in its critical period- decisions, e.g. on the composition of postsynaptic receptor fields. We have added a supplementary figure (Supplementary Figure 2) with some key manipulations to show these results.

Regarding concurrent manipulation of motoneurons and muscles, for example by exposing animals to 32ºC, the effects and severity are the same as when only one of these cells is manipulated, suggesting that there are no additive effects.

For a more detailed response, please see above – Reviewer 1.Q5

*2)	The main figures focus on HIF-1α in muscles with some evidence from the supplement that HIF-1α may also function in motoneurons? Can this data be elevated from the supplement to the main paper and also expanded upon? It is helpful to know if the same downstream effector occurs pre- and postsynaptically.*

Yes, the same pathways apply both in muscles and motoneurons. We have followed your suggestion and have added data on HIF-1α in motoneurons to the main figure (Figure 5C-E) and expanded upon it to better illustrate that the same downstream effectors are involved both pre- and postsynaptically.

*3)	Specifically regarding the change in active zone number: do ROS manipulations alter active zone density? The changes show an increase in bouton number, so naturally, it would be expected if active zone number scales with bouton number (as has been shown developmentally), then this would naturally occur. Therefore, this is expected - unless there is some shift in active zone density, which should be examined.*

We agree. Generally, bouton number and active zone number scale together. The way to demonstrate this, as the reviewer mention, is by analysing active zone density. We did this, focused on key manipulations, now included in Figure 3F-G*.*

*4)	When Ndi1 is expressed in DA1 at 25 degrees, there is an induction of synaptic changes during the critical period? If this manipulation is done at 32 degrees, do the two enhance each other? Or do they appear similar to a single manipulation? This would be helpful in ensuring that (at least genetically), they function in the same oeuvre.*

Following the reviewer's question, we tested whether mis-expressing Ndi1 in muscle DA1 or in the aCC motoneuron induces bigger synaptic changes compared when carried out at 32ºC *vs* 25ºC critical period temperature experiences; i.e. if there might be an additive effect. We found no significant differences, compatible with the model that 32ºC heat stress signals primarily or entirely via RET-generated ROS (mimicked by Ndi1 gain of function). We have included these results in figures 2C-E and 3E-F to address this point.

*5)	The authors use multiple methods to regulate HIF-1α levels. Are there suitable reagents to examine HIF-1a levels to validate these methods in cells examined?*

Following the reviewer's comments, we visualised endogenous HIF-1*α* tagged with GFP. We observed that exposure to 32ºC, as well as mis-expression of Ndi1 cause an increase in cytoplasmic HIF-1*α*::GFP and translocation to the nucleus, both in muscles and in motoneurons (Figures 4B and 5B)

*Minor Issues:*

*1)	Though I understand the concept of Figure 5B, it would be very challenging for a non-physiologist to see the phenotype the authors are describing? Could you consider a different way of presenting? Perhaps picking one specific current and showing the results? Are the changes significant? Do they trend?*

To address this concern, we have revised Figure 5B (now 5F). Specifically, we have now included a representative trace showing how the firing pattern of an aCC motoneuron changes with increasing current injection. In addition, we have annotated the graph with asterisks and “ns” (not significant) to clearly indicate which comparisons are statistically significant. These updates are intended to make the data more intuitive and to highlight the key differences in neuronal excitability due to heat stress and genetic manipulations during the embryonic critical period.

*2)	Though Table 1 is helpful, strain source data should be in the first Materials and Methods section. It would be much easier that way to determine provenance of the reagents used.*

We have updated the text according to the reviewer's suggestion.
